# Conservation and variation in the region of the *Theileria parva* p104 antigen coding gene used for PCR surveillance of the parasite

**DOI:** 10.1007/s00436-023-07838-y

**Published:** 2023-04-21

**Authors:** Isaiah Obara, Peris Makori, Kgomotso P. Sibeko, Richard P. Bishop, Ard M. Nijhof, Micky Mwamuye

**Affiliations:** 1 Department of Veterinary Medicine, Institute for Parasitology and Tropical Veterinary Medicine, Freie Universität Berlin, Berlin, Germany; 2 Department of Veterinary Medicine, Veterinary Centre for Resistance Research, Freie Universität Berlin, Berlin, Germany; 3 University of Eldoret, Eldoret, Kenya; 4 Vector and Vector-Borne Disease Research Programme, Department of Veterinary Tropical Diseases, Faculty of Veterinary Science, University of Pretoria, Pretoria, South Africa; 5 Department of Veterinary Microbiology and Pathology, Washington State University, Pullman, WA USA; 6 Department of Environment and Natural Resource Management, Africa Nazarene University, Kajiado, Kenya

**Keywords:** East Coast fever, *Theileria parva*, p104 antigen gene, Muguga cocktail

## Abstract

The range of the protozoan parasite *Theileria parva*, which causes East Coast fever in cattle, has been expanding to countries where it has not previously been detected, as a result of cross-border domestic cattle movement. Countries where *T. parva* has not previously been observed until recently include Cameroon and South Sudan. This raises the issue of the conservation of the p104 antigen gene, on which the nested PCR assay that is widely used for *T. parva* surveillance in the blood of infected cattle is based. We sampled 40 isolates from six countries widely distributed across the geographical range of the parasite, including eastern, central and southern Africa, for p104 sequence polymorphism. These included parasites from both domestic cattle and the Cape buffalo (*Syncerus caffer*) wildlife reservoir. The most frequent allelic variants were present in cattle transmissible isolates from multiple widely separated geographical regions in Zambia, Uganda, Kenya, Tanzania, Rwanda and South Africa. These frequent p104 variants were also present in the three component stocks of the Muguga cocktail used for the infection and treatment live immunisation procedure to control *T. parva* in the field. Other isolates exhibited unique alleles. This includes some of the p104 sequences from Cameroon, which is outside the known range of the *Rhipicephalus* tick vector and whose origin is therefore unclear. The nested primer oligonucleotides used to generate the amplicons were universally conserved in cattle-derived parasites and a majority of buffalo-derived isolates across the geographical range of the parasite. However, some rare South African buffalo–derived isolates exhibited one or two mismatches with the primer sequences. It therefore remains possible that some p104 alleles may be so divergent that they do not amplify with the current diagnostic primers and are not detectable in surveys, hence the need for increasing knowledge of genetic heterogeneity of diagnostic targets. There was no evidence for positive selection among those p104 mutations that resulted in residue changes. Importantly, the data indicate that the p104-based PCR detection assay should be effective across the majority of the range of *T. parva*, and if the one or two mismatches are shown in future to result in the primers annealing less efficiently, then the assay can be further improved by introduction of degenerate bases to enable amplification of the less frequent South African buffalo–derived variant p104 genes.

## Introduction

The apicomplexan *Theileria parva* immortalizes bovine lymphocytes resulting in a severe disease known as East Coast fever (ECF) in susceptible cattle, particularly exotic breeds. The disease results in an estimated one million cattle deaths annually in eastern, central and southern Africa (Norval et al. 1992) and is endemic in 12 countries extending from South Sudan to southern Africa (Irvin and Morrison 1987). Furthermore, unregulated transboundary movements of cattle with subclinical long-term infections is thought to be responsible for the spread of *T. parva* from endemic areas north into Southern Sudan (Malak et al. 2012; Marcellino et al. 2017) and west into Cameroon (Silatsa et al. 2020). Cameroon, the central African country located along a major cattle trade route between eastern and western Africa countries, is unique in the sense that *T. parva* was recently discovered there for the first time in asymptomatic cattle in four out of the five major agro-ecological zones, yet *R. appendiculatus*, the main tick vector, has not been reported. The presence of *T. parva* infections among cattle in Cameroon and the risk of spread of the infection to additional countries, such as the Central African Republic, Southwest Ethiopia and Nigeria as a result of movements of cattle with long-term asymptomatic infections, indicate that *T. parva* can be considered an emerging transboundary pathogen.

The occurrence of *T. parva* infections in previously undocumented geographical locations has increased demand for robust testing procedures to support epidemiological studies on prevalence and distribution. The most widely used serological test utilises an indirect ELISA to detect antibodies against the polymorphic immunodominant molecule (PIM), a protein antigen expressed by *T. parva* schizonts and sporozoites (Katende et al. 1998). The interpretation of the PIM-based indirect ELISA involves expressing the test sera optical density score as a percentage of the positive control serum. However, the assay can sometimes result in false positive or inconclusive results. Consequently, validation using molecular methods often becomes necessary.

The reverse line blot (RLB) hybridisation assay is a sensitive technique for detection of tick-borne pathogens, such as *Babesia bovis* that multiply in erythrocytes, whereas for *T. parva*, RLB percentage positivity is likely to represent a minimum figure because most multiplication of *T. parva* occurs in T cells, which are several orders of magnitude less prevalent than erythrocytes in blood. Given this quantitative bias, the overall percentage of cattle infected with *T. parva*, as assessed using RLB, is typically likely to be an underestimate.

More sensitive surveillance based on detection of *T. parva* genomic DNA became possible with the development and evaluation of a nested set of primers targeting the gene sequence of the *T. parva* 104 kDa (p104) rhoptry antigen (Odongo et al. 2010). However, currently there is a lack of information regarding p104 polymorphisms over the entire geospatial distribution of the parasite.

*Theileria parva* diversity has primarily been assessed using sequence polymorphism in the so-called Tp CD8 + T cell target antigen genes (Graham et al. 2006) and by analysis of a panel of variable number tandem repeat sequences (VNTRs) initially developed by Oura et al. (2003). In addition, analysis of single nucleotide polymorphisms (SNPs) through next-generation whole genome sequencing is emerging as a tool for the future (Hayashida et al. 2013; Henson et al. 2012) with the ultimate goal of performing population genomics studies. A recent study from Cameroon (Silatsa et al. 2020) surprisingly revealed seven p104 genotypes, contrasting with earlier data indicating widespread conservation in the section of the gene used for PCR-based diagnostics (Skilton et al. 2002). Some of the less frequent variants detected in the Cameroonian study had not been described previously. Only two of these variants are present in the complete genome sequences of the Muguga cocktail component stocks used for the infection and treatment immunisation (ITM), while the most frequent genotype was identical to that of the vaccine stock *T. parva* Marikebuni that was tested on several farms in the western Kenya highlands (Wanjohi et al. 2001). Given that this data indicates that additional p104 genotypes are identifiable in cattle, there is therefore a need to consider whether or not p104 polymorphism is extensive in the endemic region and can potentially impact the reliability of the PCR assay.

We report a multi-regional characterisation of the diversity present within the *T. parva* p104 gene and assess whether any genetic variation is likely to result in a discrepancy between the prevalence indicated by p104 PCR and the true presence of *T. parva* infections among cattle and buffalo in the field.

## Materials and methods

### *T. parva* isolates

The study utilized a range of *T. parva* isolates available at the Institute of Parasitology and Tropical Veterinary Medicine, Freie University Berlin and also isolates recently obtained from the field. We also analysed a further 26 near full-length p104 sequences from seven South African *T. parva* isolates obtained through a recent independent study. These isolates were selected from a collection of 111 isolates for which PIM PCR–RFLP profiles had been determined. To maximize capture of isolate diversity, the selection focused on samples that were representative of the different PCR–RFLP profile clusters. These comprised six buffalo-derived *T. parva* isolates from Kruger National Park (KNP) in Mpumalanga province (KNPAB7, KNPB10, KNP W8, KNP Y4) and Hluhluwe-iMfolozi Park in KwaZulu-Natal province (HIP 05, HIP 19). A cattle-derived isolate from Bloemfontein, Free State Province (BloeB) was also selected (Sibeko et al. 2011).

For all samples used in this study, their origin, the material used for DNA extraction and where available, the relevant reference for archived samples is provided in Table 1.

**Table 1:** Samples used in this study and details of their origin. Cattle blood was used for DNA extraction from all field samples

No	Sample	Origin	Material used	Reference
1	Ser — (Serengeti)	Tanzania	*T. parva* tick stabilate	Young and Purnell (1973)
2	Manyara buffalo	Tanzania	*T. parva* tick stabilate	Schreuder et al. (1977)
3	Pug — (Pugu)	Tanzania	Cell culture	Uilenberg et al. (1976)
4	M13 — Monduli	Tanzania	Blood	-
5	M14 — Monduli	Tanzania	Blood	-
6	M15 — Monduli	Tanzania	Blood	-
7	M36 — Monduli	Tanzania	Blood	-
8	M29 — Monduli	Tanzania	Blood	-
9	M16 — Monduli	Tanzania	Blood	-
10	M136 — Monduli	Tanzania	Blood	-
11	M141 — Monduli	Tanzania	Blood	-
12	Bol — (Boleni)	Zimbabwe	*T. parva* tick stabilate	Lawrence and Mackenzie (1980)
13	Entebbe	Uganda	Cell culture	Robson et al. (1978)
14	Sat — (Satinsyi)	Rwanda	*T. parva* tick stabilate	-
15	B11 — (Busia)	Kenya	Blood	-
16	B12 — (Busia)	Kenya	Blood	-
17	Mug — (Muguga)	Kenya	Tick salivary glands	Brocklesby et al. (1961)
18	Kia — (Kiambu)	Kenya	Tick salivary glands	Irvin et al. (1974)
19	Mar — (Marikebuni)	Kenya	*T. parva* tick stabilate	Irvin et al. (1983)
20	N4 — (Marula)	Kenya	Blood	-
21	M30 (Marula)	Kenya	Blood	-
22	N62 Marula	Kenya	Blood	-
23	N11 Marula	Kenya	Blood	-
24	N7 Marula	Kenya	Blood	-
25	N75 Marula	Kenya	Blood	-
26	N25 Marula	Kenya	Blood	-
27	N81 Marula	Kenya	Blood	-
28	N49 Marula	Kenya	Blood	-
29	N40 Marula	Kenya	Blood	-
30	N44 Marula	Kenya	Blood	-
31	N12 Marula	Kenya	Blood	-
32	N47 Marula	Kenya	Blood	-
33	Ond — (Onderstepoort)	South Africa	*T. parva* tick stabilate	Neitz (1948)
34	KNPAB7 - Kruger National park	South Africa	Blood	Sibeko et al. (2011)
35	KNPB10 - Kruger National park	South Africa	Blood	Sibeko et al. (2011)
36	KNP W8 - Kruger National park	South Africa	Blood	Sibeko et al. (2011)
37	KNP Y4 - Kruger National park	South Africa	Blood	Sibeko et al. (2011)
38	HIP 05 - Hluhluwe-iMfolozi Park	South Africa	Blood	Sibeko et al. (2011)
39	HIP19 - Hluhluwe-iMfolozi Park	South Africa	Blood	Sibeko et al. (2011)
40	BloeB - Bloemfontein	South Africa	Blood	Sibeko et al. (2011)

The Marula blood samples were collected as part of a field study undertaken to explore protection afforded to immunized animals exposed to *T. parva* challenge as described in Bishop et al. (2015).

**Fig. 1:**
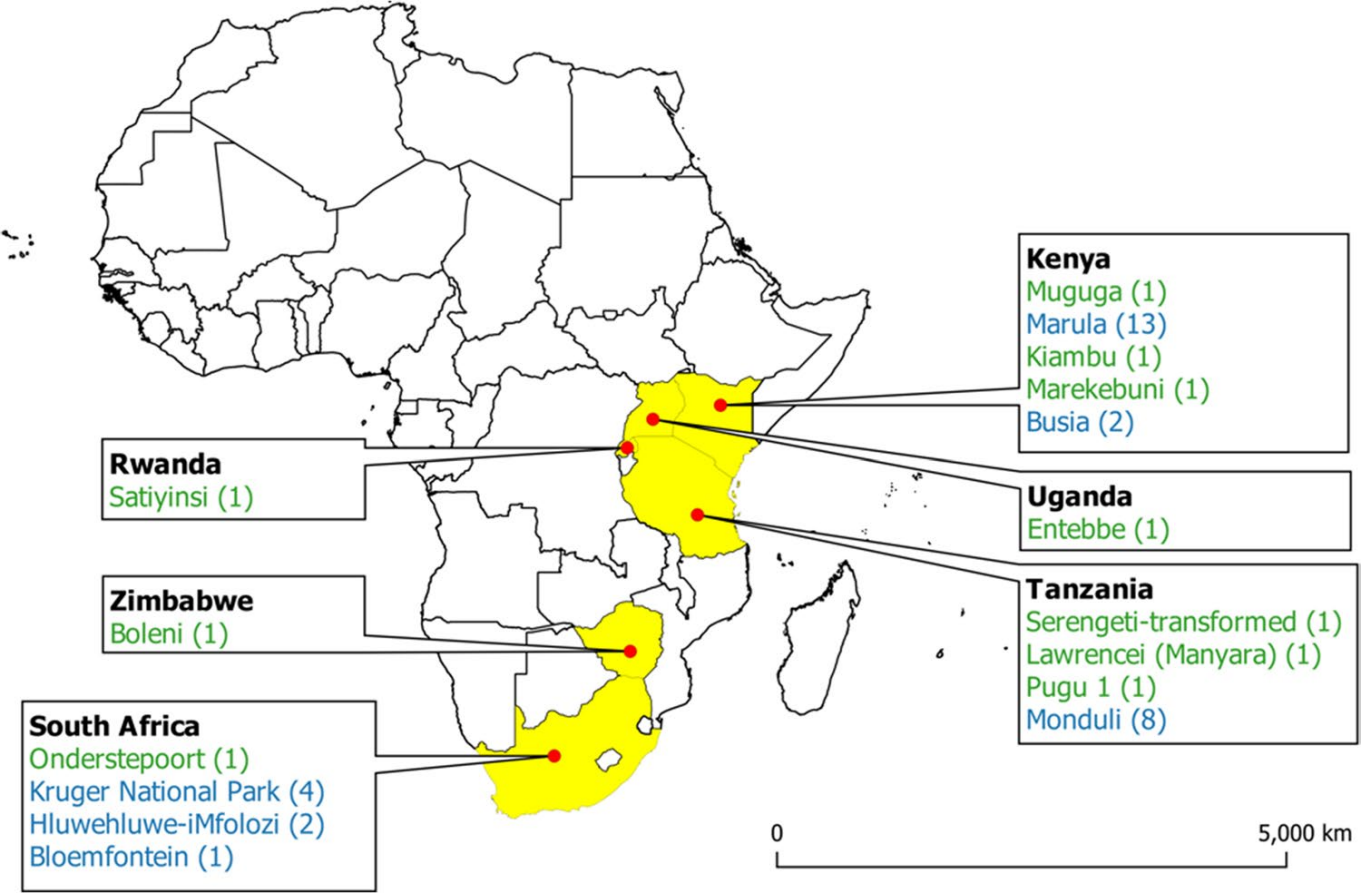
Map showing origin of *T. parva* samples used in this study. The number of isolates from each region analysed is shown in brackets. The blue font represents field samples

Figure 1 shows the geospatial distribution of the parasite isolates analysed.

### Genomic DNA extraction and amplification of the *T. parva* p104 gene

Genomic DNA was extracted from blood, frozen ground-up tick supernatants (GUTS), salivary glands and cell cultures using the Qiagen DNeasy Blood and Tissue Kit according to the manufacturer’s instructions and used as template for amplification of the *T. parva* p104 gene as previously described (Skilton et al. 2002).

Briefly, the use of oligonucleotide primers IL3231 5′-ATTTAAGGAACCTGACGTGACTGC-3′ (forward) and IL755 5′-TAAGATGCCGACTATTAATGACACC-3′ (reverse) permitted the amplification of a 496-bp fragment of the p104 gene. The amplifications were carried out using the Phusion High-Fidelity PCR reagents (Thermo Scientific) in 50 μL reaction volumes composed of 20 ng of DNA, 1 × Phusion HF buffer, 200 μM of each dNTP, 0.5 μM of forward and reverse primer and 1 unit of Phusion DNA polymerase. The PCR cycling conditions were 94 °C for 1 min, 40 cycles of 1 min at 94 °C, 1 min at 60 °C and 1 min at 72 °C, followed by a final elongation period of 2 min at 72 °C. Known positive and negative controls were included in each run. The amplification products were visualized by UV trans-illumination in 1.5% TAE-agarose gels stained with GRGreen (Excellgene, Monthey, Switzerland).

### Analysis of p104 polymorphisms

The amplicons were purified using the Zymoclean Gel DNA Recovery Kit before bidirectional Sanger sequencing. Editing and assembly of chromatograms for downstream analyses (variant calling and assessing evidence of selection) were accomplished using Geneious Prime 2021.

### Assessing evidence of selection in the *T. parva* p104 gene

We used CODEML from the PAML4 package (Yang 2007) to evaluate if there were positions in the *T. parva* p104 gene encoding residues that exhibited an excess of non-synonymous (dN) relative to synonymous substitutions (dS) in order to see whether there was evidence for positive selection for amino acid substitutions. Since models of nucleotide substitution can bias the accuracy of the phylogenetic inference, we utilized both the likelihood scores and estimated model parameters for Akaike information criterion (AIC) selection of the best-fit model from the candidate set of nucleotide substitution models. The following models were evaluated: M1a — two discrete categories, one for purifying selection where *ω* < 1, and the other for neutral selection where *ω* = 1 (Nielsen and Yang 1998; Yang et al. 2005); M2a — an extension of M1a model, with an additional category for positive selection where *ω* > 1 (Nielsen and Yang 1998; Yang et al. 2005); M7 — a continuous beta distribution of *ω* restricted to the interval (0;1), no positive selection allowed (Yang et al. 2000); and M8 — extension of M7 model, with additional, discrete category for positive selection (Yang et al. 2000). Akaike weights were used to evaluate model fit, and whenever the best-fit model is M2a or M8, sites under positive selection are determined through the Bayes empirical Bayes (BEB) approach.

To construct the input tree files used to run CodeML, we selected the best-fitting nucleotide substitution model based on the Akaike information criterion corrected for small sample size (AICc). The parameters included the following: nucleotide substitution rate parameters, equal or unequal base frequencies (+ F), a proportion of invariable sites (+ I) and rate variation among sites (+ G). Likelihood calculations for the nucleotide substitution models were performed with PhyML_3.0_linux6 (Guindon et al. 2010). All the model evaluation steps described above were implemented in jModelTest 2.1.10 (Darriba et al. 2012). Maximum-likelihood tree-search algorithms were implemented in PAUP 4.0 beta version using the parameter estimates for the best-fit model identified as described above (Swofford 2003).

## Results and discussion

### p104 gene sequence variation from multiple geographically separated regions revealed limited polymorphism which does not correlate with geographic origin

The p104 antigen is the most frequently used target gene for *T. parva* detection and surveillance. The assay is based on a nested PCR procedure designed to provide enhanced sensitivity for detection of *T. parva* genomic DNA in cattle and buffalo blood, using an outer primer pair for the first amplification, and an additional set of inner primers for the second amplification. In the present study, the outer primers and DNA extracted from a range of *T. parva* isolates (*n* = 40) obtained from multiple geographically separated areas in the endemic region were used for the amplification of the p104 gene. This study revealed 36 p104 genotypes with a mean pairwise identity at the nucleotide sequence level of 97.3%.

Although the frequently observed p104 variants were present in the three component stocks of the Muguga cocktail used for the ITM live immunisation procedure, the data is consistent with the possibility that parasites with similar p104 variants to Muguga cocktail have been circulating naturally in the field, rather than disseminated following ITM deployment. This is because the most frequent p104 alleles were also present in isolates from multiple geographically widely separated regions in Zambia, Uganda, Kenya, Tanzania, Rwanda and South Africa (Table 2), whereas the Muguga cocktail version of ITM has only been deployed extensively in Tanzania and Kenya. Only small-scale vaccination trials using the Muguga cocktail have been undertaken in Uganda. Vaccination campaigns in Zambia, use locally isolated stocks, which provides a successful alternative to the use of the trivalent Muguga cocktail version of ITM and the frequent p104 genotypes also match those present in the Chitongo and Katete vaccine stocks (Table 2). The most frequent p104 variants also match those in the Marikebuni vaccine developed by the National Veterinary Research Institute in Kenya, which has not been sufficiently widely used for ITM in the field to provide support for the vaccination dissemination hypothesis. It is also relevant to note that analysis of variation in the *T. parva* sporozoite surface antigen p67 in South African field isolates has also shown that alleles similar to those of the *T. parva* Muguga stock were circulating naturally in the field in KwaZulu-Natal Province (Sibeko et al 2011).

**Table 2:** p104 alleles, frequency and sharing among the isolates used in this study

Accession	Allele frequency (No. of isolates)	Isolates sharing allele
MZ798150	12	Chitongo (Zambia), Marikebuni (Kenya), Iganga01 (Uganda), Budako02 (Uganda), Gomba02 (Uganda), Katete (Zambia), Satinsyi (Rwanda), Kiambu (Kenya), Busia (Kenya), Marula — 25, 30, 47(Kenya)
MZ798149	10	Muguga (Kenya), Onderstepoort (South Africa), Serengeti (Tanzania), Monduli — 13,15,16,29,36,136,141 (Tanzania)
MZ798151	3	Koupa-Matapit (Cameroon), Pugu (Tanzania), Manyara (Tanzania buffalo)
MZ798152	2	BuffaloZ5E5 (Zambia), Marula 12 (Kenya)
MZ798153	1	Marula 75 (Kenya)
MZ798154	1	Marula 7 (Kenya)
MZ798155	1	Marula 44 (Kenya)
MZ798156	1	Marula 11 (Kenya)
MZ798157	1	Marula 40 (Kenya)
MZ798158	1	Boleni (Zimbabwe)

Previous molecular epidemiology and population genetics studies using polymorphic variable number tandem repeat (VNTR) sequences and nucleotide sequencing of genes that are targets of bovine CD8 + T cell responses have also demonstrated that parasite genetic diversity does not correlate with geographic origin (reviewed in Bishop et al. 2020). However, it is also important to note that in the present study, the analysis of all sequences highlights that South African buffalo–derived *T. parva* have p104 alleles that are distinct from other buffalo- and cattle-derived *T. parva*. This finding is consistent with genome and other antigen gene sequence data (Maboko et al. 2021; Sibeko et al. 2011) and is discussed in the next section with respect to single-base mismatches within the p104 primer target sequences.

**Fig. 2:**
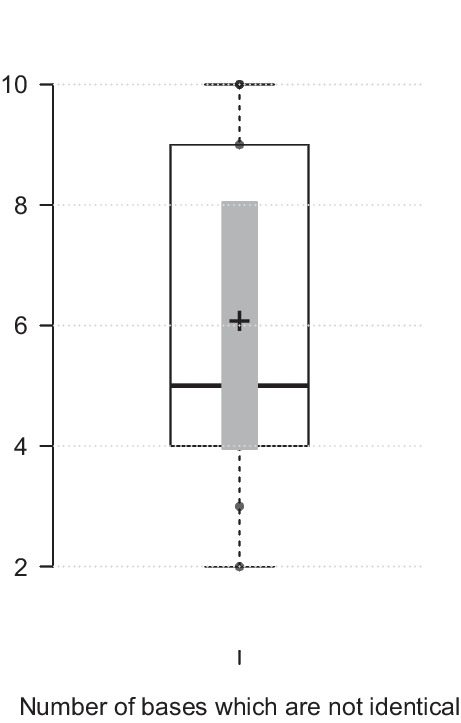
Base pair differences among ten p104 genotypes. Centre lines show the medians; box limits indicate the 25th and 75th percentiles as determined by R software; whiskers extend 1.5 times the interquartile range from the 25th and 75th percentiles; outliers are represented by dots; crosses represent sample means; bars indicate 95% confidence intervals of the means. *n* = 10 sample points

As regards sequence variation across the 496-bp amplicon, the mean number of bp differences between the genotypes was 6 (± 2.2 SD; range = 2–10; Median = 5). This limited variation is illustrated by the box plot in Fig. 2 that shows the distribution of base pair differences among the p104 alleles in the isolates studied herein.

### Single-base mismatches within the primer target sequences in rare South African buffalo–derived *T. parva* isolates

The African buffalo is the primary host for *T. parva* and is distributed across sub-Saharan Africa throughout the region where *Rhipicephalus appendiculatus*, the main tick vector of *T. parva*, occurs, but in contrast to the deadly disease suffered by cattle, buffalo remain asymptomatic. However, buffalo represent a reservoir of *T. parva* for transmission to cattle and are therefore an important consideration in assessing the extent of conservation of the *T. parva* p104 antigen gene primers used for PCR surveillance of the parasite.

Studies of genotypic diversity of *T. parva* populations in cattle and buffalo have shown that buffalo harbour parasites of much greater diversity than those in cattle and are almost always infected with multiple strains. The known diversity of *T. parva* in buffalo (reviewed by Bishop et al. 2004) and the problems with breakthroughs in ITM-vaccinated animals in areas with a cattle-buffalo interface support the suggestions that *T. parva* strains transmissible between cattle represent a subset of the overall *T. parva* population found in buffalo.

**Fig. 3:**
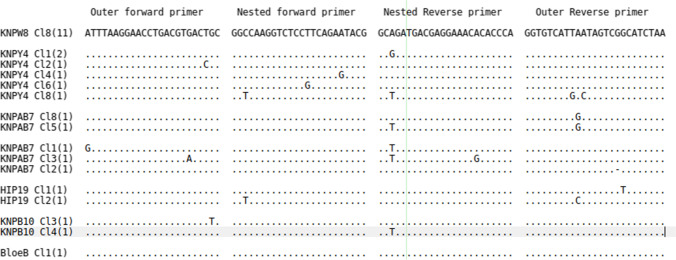
Primer matches to South African buffalo–derived *T. parva* p104 sequences

Recent studies examining the genome and antigen gene sequences of South African buffalo–derived *T. parva* have drawn the important conclusion that they are distinct from other buffalo- and cattle-derived *T. parva* (Maboko et al. 2021; Sibeko et al. 2011). Since surveillance in areas where buffalo are present in South Africa will require assays that are effective for the range of genetic variants, we have used 26 near full-length p104 sequences recently generated from seven samples representative of South African buffalo–derived field isolates (Sibeko et al. 2011), to assess the extent of conservation of the primers used for PCR surveillance. The analysis shows that the p104 primers target sequences are relatively well conserved in South African buffalo–derived isolates as no mismatches were detected in the most frequently observed p104 variant. However, multiple alignments (Fig. 3) showed one or two mismatches within the primer target sequences in some of the rare South African buffalo–derived isolates.

This may still be a rather limited picture, and the impact of the one or two mismatches in the primer target sequences on the performance of the p104 assay in the detection of rare variant, so far identified only in Cape buffalo, remains to be determined. One possibility is that the mismatches will have little effect on assay performance, and similar detection efficiencies will be observed. It may also be the case that primers may anneal less efficiently. In the latter case, the assay can potentially be improved to accommodate increasing knowledge of genetic heterogeneity of diagnostic targets by introduction of degenerate bases to allow effective amplification.

### The phylogenetic relatedness inferred from the p104 sequences does not correlate with the geographical origin of the isolates

**Fig. 4:**
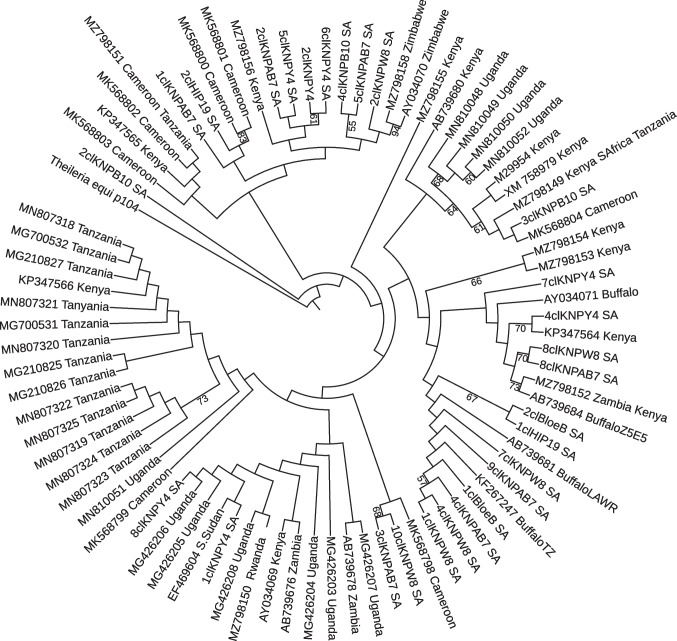
Maximum likelihood tree depicting the phylogenetic relationships of *Theileria parva* p104 sequences. The dataset includes (i) p104 allelic variants derived from the isolates investigated in the present study; (ii) p104 sequences from earlier field studies widely distributed across the geographical range of the parasite, including eastern central and southern Africa. These included parasites of both cattle and Cape buffalo wildlife reservoir origin. Numbers on the branches refer to bootstrap percentages above 50% based on 1000 replicates with *Theileria equi* p104 gene used as an outgroup

Phylogenetic inference from the p104 sequence data was based on a maximum-likelihood algorithm. Our dataset for phylogenetic inference composed of the following: (i) p104 allelic variants derived from the isolates genotyped herein, (ii) eight p104 genotypes recently described in Cameroon. Two hypotheses have been advanced about the presence of *T. parva* infections among cattle in Cameroon in the absence of the main tick vector *Rhipicephalus appendiculatus*. The first is the importation of cattle that are naturally infected or have been ITM vaccinated since Cameroon is located on a major cattle trade route between eastern and western Africa countries and therefore connected to ECF endemic countries. This hypothesis is strengthened by similarity in the amino acid sequences of *T. parva* p104 and Tp1 between Cameroonian, East African and live vaccine stocks in the case of some, but not all, Cameroonian isolates. The second hypothesis, which is considered less likely, is that *T. parva* may be actively transmitted in the field by other tick vectors, since the principal field vector has not been reported in Cameroon. It is important to emphasize that these hypotheses are speculations, and further longitudinal studies are needed for a better understanding of *T. parva* infections in Cameroon; (iii) 26 p104 sequences were generated from a representative sample of South African buffalo and cattle-derived field isolates*.* These representative isolates were selected from a collection of 111 isolates taking into account the PIM and PCR–RFLP profiles87; (iv) reference p104 sequences were distributed across the geographical range of the parasite available in the GenBank. The AIC ranking of the models resulted in the most support for the K80 + I model (Akaike weight = 0.239072). The likelihood scores, model selection criteria and numerical values for model parameters are summarized in Table 3, and the resulting tree is shown in Fig. 4.

**Table 3:** AICc ranking of a candidate set of nucleotide substitution models and parameter estimates for the model with most support

Model	− lnL	K	AICc	Delta	Weight	cumWeight
K80 + I	595.59456	68	1371.874834	0.000000	0.239072	0.239072
HKY + I	590.89823	71	1373.187764	1.312930	0.124002	0.363075
JC + I	598.37941	67	1373.943654	2.068820	0.084975	0.448050
TPM1 + I	595.10147	69	1374.423036	2.548201	0.066864	0.514914
TrNef + I	595.20330	69	1374.626696	2.751861	0.060391	0.575305
TPM2 + I	595.29533	69	1374.810756	2.935921	0.055081	0.630386
F81 + I	593.59783	70	1374.984122	3.109287	0.050508	0.680893
TPM3 + I	595.52627	69	1375.272636	3.397801	0.043723	0.724616
TPM2uf + I	590.27243	72	1375.573986	3.699152	0.037607	0.762223
TrN + I	590.31083	72	1375.650786	3.775952	0.036190	0.798413

* − lnL* negative log likelihood, *K* number of estimated parameters, *AICc* corrected Akaike information criterion, *delta* AICc difference, *Weight* AICc weight, *cumWeight* cumulative AICc weight

It is relevant to note that our understanding of the molecular epidemiology and population genetics of *T. parva* has advanced significantly in recent years (reviewed in Bishop et al. 2020). In particular, evidence is accumulating, based on polymorphic variable number tandem repeat (VNTR) sequences and nucleotide sequencing of genes that are targets of the CD8 + T cell responses from multiple geographically separated regions, that parasite diversity does not correlate with geographic origin. 

Next, we used CODEML from the PAML4 package to evaluate if there are positions in the p104 sequences coding for residues that show an excess of non-synonymous (dN) over synonymous substitutions (dS). dN/dS (*ω*) > 1 is the hallmark of positive selection for amino acid substitutions. Models that were fitted to the data included the following: M1a — two discrete categories, one for purifying selection, where *ω* < 1, and the other for neutral selection where *ω* = 1; M2a — an extension of M1a model, with an additional category for positive selection, where *ω* > 1; M7 — a continuous beta distribution of *ω* restricted to the interval (0; 1), no positive selection allowed; and M8 — extension of M7 model, with additional, discrete category for positive selection. Likelihood ratio tests were used to evaluate model support. This analysis did not reveal any sites with higher rates of non-synonymous to synonymous nucleotide substitutions than expected under neutral evolution. Both M2a and M8 models used similar numbers of parameters and the same sites under selection were identified, albeit, not supported by the BEB analysis as depicted by < 95% posterior probabilities of positive selection. A summary of the selection results is shown in Table 4.

**Table 4:** Summary of selection results showing model parameter estimates and positively selected sites under the M2a and M8 models using Bayes empirical Bayes (BEB) analysis

Model	K	LnL	Parameter estimates				Positive sites
			*ω*	p0	p1	p2	
M2a	68	− 345.2150	2.25648	0.74700	0.00000	0.25300	46 A(0.633), 51 A(0.516), 52 S(0.581)
M8	68	− 345.2150	2.25648	0.74700	0.00500	0.25300	46 A(0.752), 51 A(0.648), 52 S(0.707)

K indicates number of estimated parameters; LnL refers to maximized log likelihoods; p0 (purifying), p1(neutral) and p2 (positive) denote the proportion of codons belonging to each site class, while *ω* represents the dN/dS for the positive selection site class only. The brackets () enclose the posterior probabilities of positive selection. The codon numbers are identified with reference to sequence AB739681

Collectively, the data shows that polymorphisms in the p104 gene are unlikely to impact the PCR procedure for detection of *T. parva* in cattle and buffalo in eastern and southern Africa, since despite the existence of greater polymorphism than was previously realized within the amplicon, the primers are typically conserved. Any potential problems related to primers annealing less efficiently to the apparently rare South African buffalo–derived isolates can potentially be remedied by introduction of degenerate bases at one or two sites within each primer. It is also important to emphasize that increasing knowledge of genetic heterogeneity of diagnostic targets is an indisputably important element of molecular diagnostics and widening out into other populations remains a priority for future research.

## Data Availability

The p104 sequences generated from this study are deposited in NCBI database under accession numbers: MZ798149–MZ798158.
